# Pseudomyxoma Peritonei Misdiagnosed as Liver Cirrhosis: A Case Report and Literature Review

**DOI:** 10.7759/cureus.57857

**Published:** 2024-04-08

**Authors:** Nayef Alkhalil, Abdullah Al Omary

**Affiliations:** 1 Gastroenterology Department, Lebanese University - Faculty of Medical Sciences, Beirut, LBN; 2 Gastroenterology Department, Rafik Hariri University Hospital (RHUH), Beirut, LBN

**Keywords:** misdiagnosis, mucinous ascites, crs & hipec, intraperitoneal chemotherapy, cytoreductive surgery, pseudomyxoma peritonei

## Abstract

Pseudomyxoma peritonei (PMP) is a rare intra-abdominal malignancy characterized by diffuse dissemination of mucinous tumor cells, leading to mucinous ascites. Accurate diagnosis is crucial for appropriate management. This report presents a case of a 55-year-old Lebanese male farmer initially misdiagnosed with liver cirrhosis who presented with progressive abdominal distension refractory to diuretics and dietary modifications. Paracentesis revealed a mucinous exudate, with subsequent clinical and histopathological examination confirming PMP. The patient was referred for further evaluation at a specialized center equipped for cytoreductive surgery (CRS) and hyperthermic intraperitoneal chemotherapy (HIPEC). This case highlights the diagnostic challenges of PMP due to its non-specific presentation, emphasizing the importance of prompt and accurate diagnosis to facilitate optimal therapeutic intervention.

## Introduction

Pseudomyxoma peritonei (PMP) is a rare intra-abdominal malignant condition characterized by disseminated mucinous tumor cells and mucinous ascites ("jelly-belly") formation [1]. It presents a diagnostic challenge due to its low incidence (two to 10 cases per million/year) and often non-specific symptoms such as abdominal pain and distension [2-4]. This case report illustrates the potential pitfalls of misdiagnosis in a middle-aged Lebanese farmer with a two-year history of progressive abdominal distention and pain. Erroneously diagnosed with liver cirrhosis and subjected to potentially harmful, ineffective treatment, his case underscores the importance of accurate early diagnosis to avoid futile interventions and facilitate definitive management strategies such as cytoreductive surgery and hyperthermic intraperitoneal chemotherapy.

## Case presentation

A 55-year-old Lebanese man, who works as a rural farmer and is known to have hypertension, presented with a two-year history of progressively worsening abdominal distension associated with mild intermittent abdominal pain and fatigue. The patient’s abdominal swelling limited his daily life activities and oral intake, so he sought medical attention. The patient was previously told that he has liver cirrhosis, without having any obvious risk factors and no convincing imaging or laboratory findings to support this claim. Subsequently, he was prescribed oral diuretics (spironolactone and furosemide) and dietary modifications (low-salt diet) without any improvement despite compliance with treatment.

**Figure 1:**
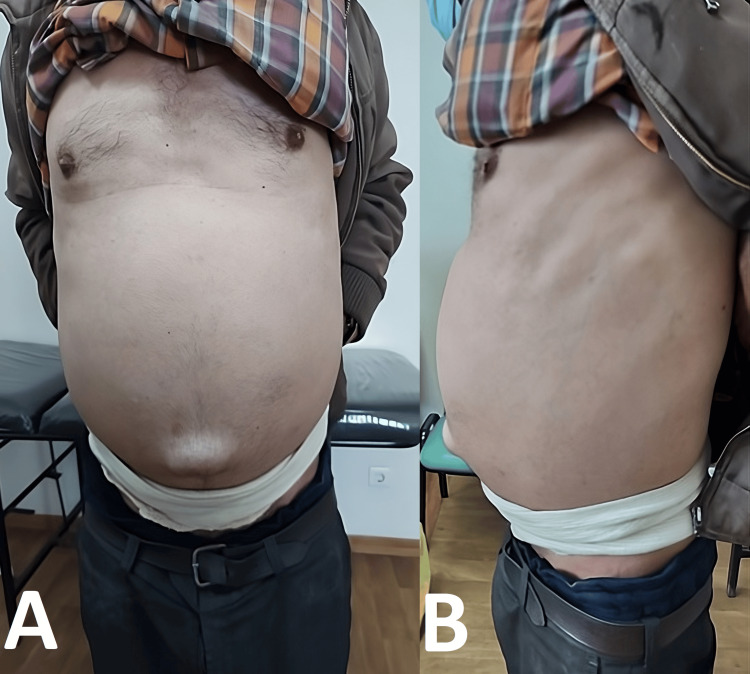
Anterior and lateral views of the patient's abdomen on physical examination Pictures A and B depict the patient's anterior and lateral views, respectively (distended abdomen with bulging umbilical hernia). The patient presented wearing a white belt (evident in the pictures) around his abdomen to support it.

On physical examination, he was conscious, cooperative, and oriented. His vital signs were within normal limits (temperature of 36.7 °C, HR of 82 beats/minute, RR of 18 breaths/minute, BP of 120/63 mmHg), and his pulmonary and cardiovascular examinations were normal. He was not icteric, and he did not have jugular venous distension. However, his abdomen was severely distended and not tender and had a bulging umbilical hernia. There was no spider telangiectasia, palmar erythema, or distended abdominal veins (Figure 1).

Abdominal ultrasound was done, and it showed large-volume ascites, a homogenous liver with irregular borders measuring 13 cm and with no visible masses within, normal spleen, kidneys, and adrenals, and no abdominopelvic lymphadenopathy. The patient was admitted for further investigation of his ascites.

Initial laboratory investigations revealed microcytic anemia with hemoglobin of 9.09 g/dL consistent with iron deficiency (reference range: 13.5-17.5 g/dL). Liver function tests, electrolytes, and coagulation parameters were unremarkable, except for elevated alkaline phosphatase (ALP) at 303 U/L (reference range: 25-100 U/L) and gamma-glutamyl transferase (GGT) at 217 U/L (reference range: 8-38 U/L), suggestive of cholestasis. Viral hepatitis markers (HBsAg and HCV antibodies) were negative. Tumor markers alpha-fetoprotein (AFP) and prostate-specific antigen (PSA) were within reference ranges, but carcinoembryonic antigen (CEA) was significantly elevated at 234 ng/mL (reference range: <2.5 ng/mL). Antinuclear antibody (ANA) testing was negative. For detailed laboratory values, please refer to Table 1.

**Table 1:** Our patient's laboratory test results

Lab Test	Result	Reference Range	Lab Test	Result	Reference Range
White blood cell (WBC) count	7,990	4,500-11,000/mm^₃^	Alanine transaminase (ALT)	25	10-33 U/L
Haemoglobin	9.09	13.5-17.5 g/dL	Aspartate aminotransferase (AST)	24	10-33 U/L
Mean corpuscular volume (MCV)	68	80-100 fL/cell	Gamma-glutamyl transpeptidase (GGT)	217	8-38 U/L
Platelet count	450,000	150,000-450,000/mm^₃^	Alkaline phosphatase (ALP)	303	25-100 U/L
Creatinine	0.81	0.6-1.2 mg/dL	Bilirubin total	0.5	0.1-1 mg/dL
Sodium	144	136-146 mEq/L	Antinuclear antibody (ANA) panel	Negative	Negative
Potassium	4.12	3.5-5 mEq/L	Hepatitis B surface antigen (HBsAg)	Negative	Negative
Chloride	101	95-105 mEq/L	Hepatitis C antibody (HCV Ab)	Negative	Negative
Bicarbonate	25.9	22-28 mEq/L	Albumin	3.47	3.4-5.5 g/dL
Ferritin	281	15-200 ng/mL	International normalized ratio (INR)	1.02	0.8-1.1
Serum iron	34	60-170 mcg/dL	Prostate-specific antigen (PSA)	0.47	0-2.5 ng/mL
Total iron binding capacity (TIBC)	220	240-450 mcg/dL	Carcinoembryonic antigen (CEA)	234	<2.5 ng/mL
Transferrin saturation	15	20-50%	Alfa-fetoprotein (AFP)	5.62	0-40 ng/mL

**Figure 2:**
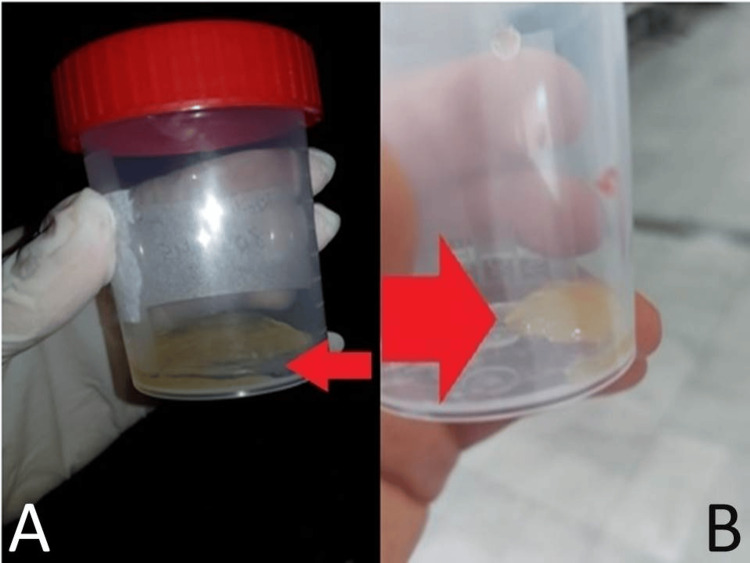
Yellowish viscous abdominal fluid aspirated by abdominal paracentesis Picture A shows the yellowish slimy semi-solid fluid (small red arrow) aspirated on the first abdominal paracentesis. Picture B shows the thick yellowish gelatinous fluid (big red arrow) aspirated on another abdominal paracentesis to obtain further diagnostic studies.

Abdominal paracentesis was performed and showed markedly yellow viscous thick fluid with jelly-like consistency (Figure 2). These findings were very unusual and raised our suspicion of PMP.

Abdominal fluid analysis showed WBCs of 10 cells/mm, RBCs of 140 cells/mm, total protein of 13,603 mg/dL, albumin of 2.1 g/dL, and LDH of 973 UI, and bacterial culture came back negative.

Cytology analysis showed mucinous material containing clusters of moderately atypical cells. Thus, a sample was sent for immunohistochemical (IHC) staining that was negative for CK7, CK20, CDX-2, TTF-1, and calretinin, stating that the present cells represent macrophages in a background of abundant mucin and the fluid was negative for malignant cells (acellular mucin). The analysis was done and confirmed at two different pathology labs.

**Figure 3:**
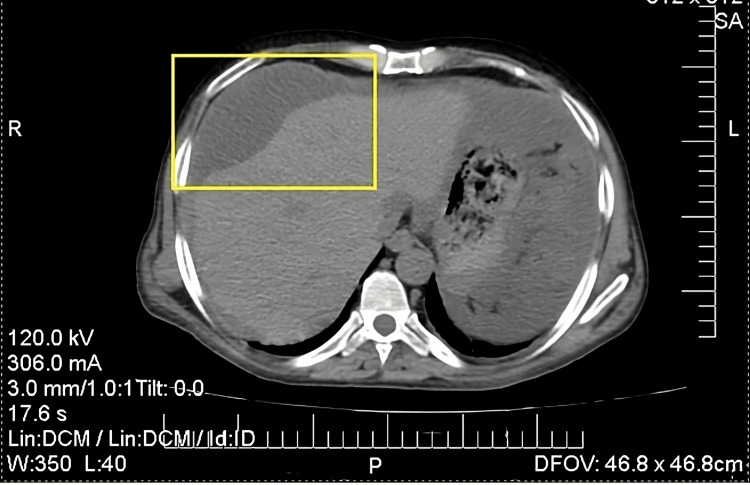
The scalloping of the liver (yellow box) caused by the thick abdominal mucus, a characteristic finding of PMP compared to usual abdominal fluid/ascites PMP: Pseudomyxoma peritonei

On admission, a CT scan of the abdomen and pelvis with IV contrast was done and showed a shrunken liver with nodular borders. There was also a diffuse severe loculated ascitic fluid showing a thickened enhanced peritoneal wall with mildly enhanced peritoneal nodularities/deposits, some of which show punctuate and curvilinear calcifications causing omental scalloping of the visceral liver (Figure 3) and splenic surfaces (omental caking). Findings were consistent with PMP.

Afterward, to identify the primary origin of the abdominal mucus, especially in light of elevated CEA level, endoscopy was performed. Gastroscopy was normal. However, the colonoscopy showed a small appendicial polyp that was removed by biopsy. Cytology report showed that this polyp is colonic mucosa with mucinous exudate that likely, in our opinion, represents the origin of the mucoid ascites.

**Figure 4:**
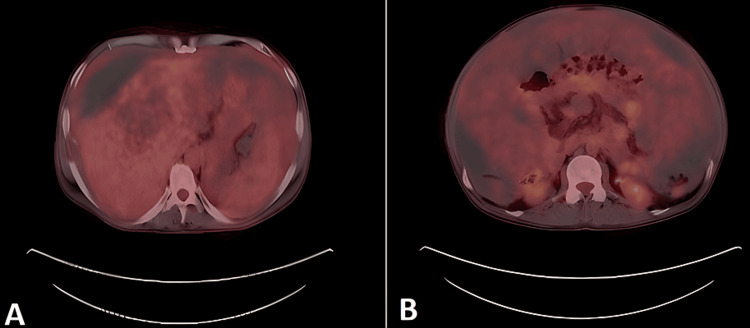
FDG-enhanced PET CT scan axial images of the patient Picture A shows the scalloping of the liver and peritoneal and omental thickening with mild increased uptake surrounding non-FDG-avid mucin. Picture B shows the bowel loops stacked in the mid-line with surrounding non-FDG-avid mucin. FDG: 18F-fluorodeoxyglucose

Then, the patient was referred to do an 18F-fluorodeoxyglucose (FDG)-enhanced total body PET scan that showed evidence of large PMP involving the abdomen and pelvis with mildly increased uptake of the peritoneum. Faint uptake is seen associated with anterior peritoneal masses (Figure 4). The skeletal system shows no suspicious uptake. Findings are compatible with PMP with multiple active omental masses that could be of appendiceal origin, with no FDG-avid lymphadenopathy.

This case report describes a middle-aged Lebanese man presenting with PMP (acellular mucin). While appendiceal origin is suspected based on clinical presentation, a definitive diagnosis regarding the primary tumor location could not be established at our facility due to limitations in diagnostic capabilities. Therefore, to ensure optimal management, the patient was referred to a specialized medical center equipped for a definitive diagnosis through laparotomy and potential cytoreductive surgery (CRS) with hyperthermic intraperitoneal chemotherapy (HIPEC) if indicated.

## Discussion

PMP, a rare clinical and pathological condition, impacts only two to 10 individuals per million globally [1,2]. Its limited occurrence qualifies it as an "orphan disease," officially recognized by the National Association of Rare Disorders (NORD) [3]. This designation stems from its estimated prevalence of less than 200,000 affected individuals in the United States (US) alone [3].

The defining characteristic of PMP is the pervasive spread of mucus-producing tumor cells within the abdomen [4]. As the mucus accumulates throughout the peritoneal cavity, it causes mucinous ascites, also called “jelly belly.” This condition, first described in 1884 by Werth et al., was initially thought to only arise from bursting ovarian cysts. However, in 1901, Frankel and his team reported a case linked to a burst appendiceal cyst, showing that PMP could stem from different sources [5].

The disorder usually develops after an appendiceal polyp bursts through the wall of the appendix and spreads mucus-producing tumor cells throughout the abdomen. These tumor cells replicate and produce a mucinous by-product that spreads through the abdomen. While the most common cause of PMP is appendicial cancer, several types of tumors (including non-cancerous tumors) can cause PMP such as the ovary, stomach, colon, pancreas, and other abdominal organs [4].

It is a misconception that females develop this disorder more frequently since males and females are both equally affected in numbers [6]. The vast majority of PMP diagnoses occur between the ages of 40 and 50, although cases have been reported as early as the late teens and later in life [6]. Currently, there is no evidence of a familial link or established risk factors associated with PMP development [6].

Our patient, a middle-aged man in his 50s, falls within the typical age range reported for this presentation. This finding aligns with the cases by Pantiora et al., both males in their late 50s [7]. However, our case diverges from Joseph et al. who reported an 86-year-old female patient with PMP [8], and Sarpietro et al. who described a 37-year-old female diagnosis [9]. These age discrepancies highlight the variable presentation of PMP, extending beyond middle age, as seen in Joseph et al.'s [8] study, and encompassing younger individuals, as noted by Sarpietro et al. [9].

The clinical presentation of PMP is primarily governed by the progressive accumulation of mucinous ascites within the abdomen, leading to increased intra-abdominal pressure [5,10]. Interestingly, a subset of patients remain asymptomatic for extended periods, with diagnosis occurring only incidentally during imaging or surgical procedures [10]. Notably, the majority of patients lack well-defined symptomatology until disease progression reaches a relatively advanced stage [5]. The most frequent presenting complaints, affecting 30-75% of both genders, are progressive abdominal distention and inguinal hernia formation [5]. Consequently, the encounter of mucoid fluid during hernia repair necessitates meticulous cytological analysis to facilitate the identification of potential occult PMP [5]. Additional commonly reported symptoms include abdominal bloating, discomfort, and intestinal obstruction, and the latter, unfortunately, represents the leading cause of mortality in this patient population [5,10].

The diagnosis of PMP is clinico-pathologic and is sufficiently established by the presence of viscous mucinous fluid, with or without neoplastic cells, in the peritoneal cavity [11]. Immunohistochemical markers can be used to determine the primary tumor site in a patient with PMP. Appendicial tumors usually express CK20, CDX2, MUC2, and MUC5AC and are negative for CK7 and CA-125 [5].

In 2016, the Peritoneal Surface Oncology Group International (PSOGI) published a written consensus on the diagnostic terminology and classification of PMP. PSGOI classified PMP into four broad categories (from least to most aggressive) [12-14]:

1) Acellular mucin: mucin lacking neoplastic epithelium and may be distant from or confined to the organ surface.

2) Low-grade mucinous carcinoma peritonei (LGMCP): low-grade cytology, few tumoral mucinous epithelium (<20% of tumor volume), and rare mitoses.

3) High-grade mucinous carcinoma peritonei (HGMCP): high-grade cytology, metastatic and invasive nature, and contains neoplastic mucinous epithelium (>20% of tumor volume).

4) High-grade mucinous carcinoma peritonei with signet ring cells (HGMCP-SRC): tumor with signet ring cell component (≥10%).

While a definitive diagnosis of PMP is mainly clinical and pathological, imaging plays a crucial role in lending support and even hinting at the source of the mucinous material. One important early radiographic finding is the unusual positioning of tumor deposits, a phenomenon known as the "redistribution phenomenon." In this phenomenon, large tumor deposits accumulate at specific pre-determined anatomical locations within the peritoneal cavity, while other areas show minimal or no involvement. These preferred sites for PMP include the greater omentum ("omental cake"), undersurface of the right hemidiaphragm, pelvis, right retrohepatic space, left abdominal gutter, and ligament of Treitz. Notably, the peritoneal surfaces of the bowel remain largely uninvolved due to their constant peristaltic motion, hindering extensive tumor implantation [15]. Scalloping of the liver, spleen, and mesentery are also characteristic findings that further strengthen the suspicion of PMP [16].

While the specific role of tumor markers in diagnosing PMP remains under debate, their elevation, particularly of CEA, CA-125, and CA19-9, can indicate PMP activity and provide valuable prognostic and post-surgical monitoring information [4]. However, their sensitivity is inconsistent, with some patients not exhibiting elevated markers despite advanced disease [5].

The definitive treatment for PMP depends on the underlying pathology. However, for appendiceal PMP, a widely accepted approach often relies on the combined approach of CRS, which involves complete resection of all macroscopic tumor deposits, followed by HIPEC to eradicate microscopic tumor cells. This approach was originally pioneered and established by Sugarbaker et al. for appendiceal PMP [17,18].

Our patient's presentation primarily involved a two-year history of abdominal distension and vague abdominal pain. This feature aligns with the case reported by Joseph et al., which described worsening indigestion and abdominal distension within a two- to three-month timeframe [8]. In contrast, the case reported by Sarpietro et al. presented with infertility and pelvic pain, while Pantiora et al. described a patient with changing bowel habits over a year [7,9]. These diverse presentations highlight the broad clinical spectrum of PMP, which encompasses gastrointestinal, genitourinary, and non-specific symptoms such as abdominal distension.

Consistent with the reported limitations of tumor markers in PMP diagnosis, our patient exhibited an elevated CEA level (234 ng/mL), while AFP and PSA remained within normal ranges. Pantiora et al. similarly described an elevated CEA (210 ng/mL) but unremarkable CA 19-9, further illustrating the variability of marker expression [7]. Conversely, Joseph et al. reported normal CEA and CA 19-9 in their case, highlighting the potential for normal levels even in advanced disease [8]. These discrepancies underscore the unreliable nature of tumor markers for definitive PMP diagnosis, necessitating a multi-modal approach incorporating clinical features, imaging, and tissue biopsy.

Finally, this case highlights the potential need for broader access to specialized surgical expertise for optimal management of PMP. While our institution provided comprehensive preoperative care, the lack of available CRS + HIPEC limited our ability to offer complete follow-up.

## Conclusions

In conclusion, PMP is a rare clinical condition associated with mucinous tumors. It has a varying clinical presentation, from being asymptomatic to abdominal pain, distension, and possibly bowel obstruction. Clinicians should maintain a high clinical suspicion of this condition in appropriate age groups and case scenarios, to avoid jumping into more common diseases that share similar clinical presentation, and expose patients to unnecessary and potentially harmful treatment options.
